# Musculoskeletal pain as the effect of internal compensatory mechanisms on structural and functional changes in body build and posture in elite Polish sitting volleyball players

**DOI:** 10.1186/s13102-022-00439-9

**Published:** 2022-03-26

**Authors:** Anna Zwierzchowska, Eliza Gawel, Diana Celebanska, Barbara Rosolek

**Affiliations:** Institute of Sport Sciences, The Jerzy Kukuczka Academy of Physical Education in Katowice, Mikołowska 72A, 40-065 Katowice, Poland

**Keywords:** Paralympic volleyball, Pelvic inclination, Spine, LBP, BAI

## Abstract

**Background:**

With the dynamic development of professional Paralympic sport, the prevalence of musculoskeletal pain and structural and/or functional disturbances in Para athletes constantly increases. The aim of the study was to evaluate the impact of internal compensatory mechanisms on selected aspects of body structure and function in elite sitting volleyball players.

**Methods:**

The study included eighteen elite sitting volleyball players (male; n = 12, female; n = 6, age; 36.0 ± 6.1, body mass; 76.6 ± 16.1, body height; 179.3 ± 0.1) from the Polish national team. Retrospective and direct participatory observation methods were used in the study. NMQ-7 was used to assess the current prevalence and location of musculoskeletal pain. The evaluation of spinal curvature and pelvic inclination was performed using a non-invasive Medi Mouse method (Idiag M360) in three different trunk positions. All statistical analyses were performed using Statistica 13.3 software package.

**Results:**

Lumbar hypolordosis was a predominant sagittal deviation of spinal curvature (n = 15;83%). Low back pain (LBP) and neck pain were the most frequent complaints (50%). Statistically significant differences in the values of thoracic kyphosis angle, pelvic inclination, and spine length (SL) in sagittal standing flexion and extension were found. However, there was no statistically significant difference in sagittal standing flexion for the lumbar lordosis angle with a simultaneous significant change in pelvic inclination (66.9°). Moreover, a tendency to interpenetration of relationships between variables that characterize (a) body structure and (b) function of the spine and musculoskeletal pain were observed. Shoulder pain correlated with SL (R = 0.6; *p* < 0.05) and body height (R = 0.5; *p* < 0.05). Pelvic inclination correlated with shoulder pain, LBP (R = 0.5; *p* < 0.05/R = 0.6; *p* < 0.01), and body trunk fat mass (R = − 0.6; *p* < 0.05).

**Conclusions:**

Trunk fat mass induces internal compensatory mechanisms to maintain optimal pelvic inclination and sagittal spinal balance. Furthermore, the level of pelvic mobility may determine musculoskeletal pain in Para athletes with lower limb impairment.

## Background

Pain is known to be one of the most frequent problems both in Olympic and Paralympic sports [1]. To achieve an elite level of sports performance, avoidance and management of musculoskeletal pain are necessary for each athlete to continue training, which is believed to be a core aspect of sports performance [2]. Sport-specific movements modify the body build and posture of athletes, contributing to both structural and functional adaptations in the athlete’s body [3]. However, the adaptations are not always necessarily beneficial as they tend to lead to muscular imbalances and cause musculoskeletal pain, which may affect both sports performance and athlete’s quality of life [4, 5].

Human body structure and function are the two intrinsic and strongly connected elements [6]. Therefore, it is difficult to indicate the primordiality of changes in the musculoskeletal system, especially in Para athletes with musculoskeletal impairments because of the disturbed body’s biomechanics. The human body always strives for balance by using compensatory strategies, even if they are not fully beneficial [4]. Gaweł et al. [4] indicated two crucial compensatory mechanisms in Para athletes internal and external. The mechanism of external compensation is known as the body’s adaptations to sport-specific movements, whereas the internal mechanism is an essential compensatory strategy due to a congenital or acquired impairment, that is interdependent mostly with biomechanical factors and body composition [4]. Nevertheless, both of the abovementioned mechanisms simultaneously disturb the intrinsic body’s biomechanics e. g. by changing the pelvis inclination or disturbing the fat mass distribution, leading to musculoskeletal pain.

The body’s structure and function are strongly related to the stability and mobility of muscles and joints [7, 8]. As the load distribution between passive and active segments of the human body in various everyday life activities is mainly determined by the spine and pelvic position [9], the levels of stability and mobility in spinal joints are crucial to avoid disturbances in body build and posture. This issue has significant importance in Para athletes with lower limb impairments as it has been reported that lower limb amputations and defects significantly impact human biomechanics [10, 11]. Another component that is intrinsically related to the body’s biomechanics is body composition i.e. fat mass and muscle mass [12]. The level, variability, and distribution of the abovementioned components depend both on endogenous (neuromuscular or musculoskeletal disorders/impairments, intracellular metabolism) and exogenous (lifestyle, physical activity) factors [5, 13, 14]. Despite the factor that disturbs the body’s structure and/or function, internal compensatory mechanisms are activated as they intrinsically maintain the body’s homeostasis [4].

A study by Zwierzchowska et al. [15] showed a relationship of the level and distribution of fat mass with deviations in spinal curvature. It was found that an inappropriate visceral fat mass affects anteroposterior spinal curvatures, mainly by deepening lumbar lordosis, which might cause low back pain (LBP) [15, 16]. Fat mass is usually assessed based on the body mass index (BMI). However it has been shown that the body adiposity index (BAI) is a more reliable marker, especially for people with disabilities, e.g. amputees [15, 17, 18].

With the dynamic development of professional Paralympic sport the prevalence of musculoskeletal pain and structural and/or functional disturbances in Para athletes constantly increases [4, 19]. To date, several hypotheses have been proposed, but this issue remains unsolved. Therefore, the aim of the study was to identify the impact of internal compensatory mechanisms onselected aspects of the body’s structure and function in elite sitting volleyball players. It was assumed that excessive trunk fat mass, overweight, and low mobility of the lumbar spine contribute to musculoskeletal pain in Para athletes with lower limb impairments.

## Methods

### Participants

The study included eighteen elite sitting volleyball players (male: n = 12, female: n = 6, age: 36.0 ± 6.1, body mass: 76.6 ± 16.1, body height: 179.3 ± 0.1, experience in sitting volleyball training (years); 8.1 ± 7.6) from the Polish national team. The inclusion criteria were as follows: (1) at least a minimal disability (MD) according to the World ParaVolley classification, (2) at least 2 years of training at an elite level (3) free from neuromuscular or musculoskeletal disorders. Table 1 provides a detailed description of the study participants.

**Table 1:** Detailed characteristics of the study participants

Participants’ characteristics (n = 18; nF = 6, nM = 12)	Mean ± SD
Age (years)	36.0 ± 6
Body mass (kg)	76.6 ± 16.1
Body height* (m)	179.3 ± 0.1*
Hip circumference (cm)	102.7 ± 10.4
Trunk fat mass (%)	29.07 ± 9.6
Waist circumference (cm)	89.4 ± 11.9
BMI in participants with lower limb deficiency (n = 14)	23.4 ± 5.1
BMI (n = 18)	25.5 ± 3.4
BAI* (%)	24.9 ± 4.1
Duration of paralympic sport-specific training (years)	8.9 ± 7.9
Duration of disability (years)	21.9 ± 10.1

Disability classification	Percentage (%)
Amputees	67
Les autres	33

n—total number of participants; nF—number of females; nM—number of males; *excluded bilateral amputation (n = 1)

*SD* standard deviation, *BMI* body mass index, *BAI* body adiposity index

The amputee group used prostheses (n = 10) or orthopedic crutches (n = 1) in the activities of daily living and locomotion. Only one athlete had a bilateral amputation above the knees and used a wheelchair in everyday life. The athletes from the Les Autres group used prostheses (n = 2), orthopedic crutches (n = 2), and no supportive equipment (n = 2).

The examinations started in December 2020 (pilot studies) [4], whereas further data were collected in March and November 2021 as a part of a project of monitoring the training process and body’s adaptations and compensations in elite Polish sitting volleyball players in different training periods. All measurements were carried out at the Jerzy Kukuczka Academy of Physical Education in Katowice, Poland. Participants were informed about the advantages and disadvantages of the research and provided written informed consent. The research protocol was approved by the Bioethics Committee for Scientific Research at the Academy of Physical Education in Katowice, Poland (No. 9/2012) and met the ethical standards of the Declaration of Helsinki, 2013. Moreover, participants were allowed to withdraw from the experiment at any moment. Furthermore, they were instructed to maintain their normal dietary and sleeping habits for 24 h before the examination.

### Measurements

Retrospective and direct participatory observation methods were used in the study. Athletes arrived at the laboratory in the morning (8–11 a.m.). The prevalence of musculoskeletal pain was first assessed. Next, the anthropometric and spinal curvature measurements were performed.

### The assessment of the prevalence of musculoskeletal pain

The Nordic Musculoskeletal Questionnaire from the last 7 days (NMQ-7) [20] was used to assess the current prevalence and location of musculoskeletal pain among sitting volleyball players, as it is known to provide useful and reliable data on musculoskeletal pain. The questionnaire has been tested for (a) validity, which showed almost identical answers concerning the clinical history of the study participants (ranging from 80 to 100%), and (b) reliability, which showed similar results (range: 78–100%) [20]. The NMQ-7 includes the following nine body parts: the neck, shoulders, upper back, elbows, wrists, lower back, hips/thighs, knees, and ankles/feet. To minimalize the subjective risk of error, the questionnaire was completed in the presence of a physiotherapist. Moreover, before completing the questionnaire, sitting volleyball players were instructed not to report phantom pain because of psychological factors that might contribute to this phenomenon [21].

### Anthropometric measurements

To assess the body height (BH), a wall-mounted stadiometer with a centimeter scale was used, including the wheelchair user who was able to stand for a short period of time without additional supporting equipment. Body mass (BM) was evaluated using a chair balance. The circumferences of hips (HC) and waist (WC) were assessed with the use of an anthropometric tape on bare skin in a lying position and according to the recommended anthropometric techniques, i.e., HC—around the greatest convexity of the gluteal muscles below the iliac ala, WC—at the midpoint between the superior iliac crest and the lowest rib [22]. Body mass index (BMI) was calculated by the standard formula (body mass [kg]/body height^2^ [cm]). However, as the majority of the study participants were characterized by a limb deficiency, a corrected BMI (based on the Brown-Fisher rate) was also computed. The proportion of total body weight assigned to different body segments was as follows: hand (1%), forearm (2%), arm (3%), head (7%), trunk (43%), thigh (12%), shank (5%), and foot (2%) [23]. The assessment of fat mass was performed with a Tanita Viscan AB-140 Abdominal Fat Analyzer, which is known to be a golden standard for individuals who cannot stand in the upright position and has 95% sensitivity and reliability. The body adiposity index (BAI) (BAI = hip circumference[cm]/(body height [m]) − 18) was also computed. BAI is believed to be an objective marker to evaluate body adiposity in both able-bodied and disabled populations [15, 17, 18].

### Spinal curvatures measurements

The evaluation of the spinal curvatures and pelvic inclination was performed based on a standard procedure similar to that used in the pilot study [4] using a non-invasive Medi Mouse method (Idiag M360), by the same two specialists (EG, DC). Medi Mouse ensures reproducibility even if two different researchers conduct the examinations [4]. All procedures were demonstrated and explained. Next, the measurements were conducted in three different trunk positions [4], i.e., sagittal standing (arms in the habitual position), sagittal standing flexion (arms in a free stance), and extension (arms crossed on the shoulders, elbows up). The measurement started with placing the Medi Mouse at the C_7_ level. Next, the device was moved at a constant speed up to the S_5_ level [4]. All measurements were automatically recorded on a computer with Idiag M360 software to collect the data on (a) the current depth of thoracic and lumbar curvatures, (b) the proper physiological values of the aforementioned curvatures based on the values obtained for the able-bodied populations similar in gender, age, BH and BM, (c) the differences between the current and proper physiological values of thoracic and lumbar curvatures, (d) the type of sagittal spinal curvature deviation, i.e., thoracic hyper/hypo kyphosis, and lumbar hyper/hypo lordosis.

### Statistical analysis

All statistical analyses were performed using Statistica 13.3 software package. Distributions, means, and standard deviations (SD) of the anthropometric characteristics (BM, BH, BL, WC, HC), indices (BMI, BAI), and spinal curvatures (TK, LL, PV, spine length, pelvic inclination) were verified (Kolmogorov–Smirnov test). The percentage of thoracic hyperkyphosis, thoracic hypokyphosis, lumbar hyperlordosis, lumbar hypolordosis of sitting volleyball players was calculated. The correlations of the anthropometric characteristics (BM, BH, BL, WC, HC), indices (BMI, BAI), spinal curvatures (TK, LL, PV, spine length, pelvic inclination), and musculoskeletal pain were verified (Spearman’s correlation). Variability of the kTH, kLL, spine length, pelvic inclination in sagittal standing, sagittal standing extension, and sagittal standing flexion was verified (Wilcoxon signed-rank test). Correlations were evaluated as follows: trivial (0.0–0.09), small (0.10–0.29), moderate (0.30–0.49), large (0.50–0.69), very large (0.70–0.89), nearly perfect (0.90–0.99), and perfect (1.0). The significance level was set at *p* < 0.05.

## Results

The quantitative and qualitative assessment of the body posture of sitting volleyball players is presented in Tables 2 and 3. It was shown that lumbar hypolordosis was a predominant sagittal spinal curvature deviation (n = 15; 83%), whereas the values of lumbar lordosis angle were normal only in two players (n = 2;11%). Simultaneously, it was found that the prevalence and location of musculoskeletal pain based on NMQ-7 were the most frequent in the lower back and neck (50%).

**Table 2:** Quantitative and qualitative characteristics of physiological spinal curvatures in the sagittal plane in three positions (sagittal standing, sagittal standing flexion, sagittal standing extension) in elite sitting volleyball players

Spinal curvatures measurements—sagittal plane	Mean ± SD (°)	Values of the spinal deviations—sagittal plane	Number and percentage (%)
kTH-sagittal standing	36.9 ± 15.8	Thoracic hyperkyphosis	7 (39%)
PV of kTH	38.3 ± 16.6		
Difference between kTH and PV	2.9 ± 3.9		
kTH-sagittal standing flexion	60.4 ± 13.9	Thoracic hypokyphosis	7 (39%)
PV of kTH	59.6 ± 15.6		
Difference between kTH and PV	3.8 ± 5.1		
kTH-sagittal standing extension	32.6 ± 15.5	Norm	4 (22%)
PV of kTH	31.5 ± 16.5		
Difference between kTH and PV	4.0 ± 4.7		
kLL-sagittal standing	15.5 ± 10.6	Lumbar hyperlordosis	1 (6%)
PV of kLL	17.6 ± 13.0		
Difference between kLL and PV	3.1 ± 4.0		
kLL -sagittal standing flexion	22.3 ± 12.7	Lumbar hypolordosis	15 (83%)
PV of kLL	22.9 ± 16.0		
Difference between kLL and PV	3.7 ± 5.1		
kLL -sagittal standing extension	24.6 ± 14.1	Norm	2 (11%)
PV of kLL	25.3 ± 14.5		
Difference between kLL and PV	3.9 ± 4.6		

*kTH* thoracic kyphosis angle, *PV* physiological values, *kLL* lumbar lordosis angle, *SD* standard deviation

**Table 3:** The prevalence (%) and locations of musculoskeletal pain based on NMQ—7

Body parts (NMQ-7)	Percentage (%)
Neck	50
Shoulders	22
Upper back	44
Elbows	17
Wrists	22
Low back	50
Hips/ties	22
Knees^a^	28
Ankles/feet^a^	22

^a^One participant did not answer because of bilateral amputation above the knees

Comparative analysis of the variability of the selected elements of the body posture during sagittal standing flexion and extension regarding sagittal standing showed statistically significant diversity in the values of thoracic kyphosis angle, pelvic inclination, and spine length. However, there was no statistically significant difference in the values of lumbar lordosis angle during sagittal standing flexion with a simultaneous significant change in pelvic inclination (66.9°)(Table 4).

**Table 4:** Variability of selected elements of the body posture in the sagittal plane in three positions (sagittal standing, sagittal standing flexion, sagittal standing extension)

The level of significance of the analyzed variable between sagittal standing and sagittal standing flexion	Sagittal standing flexion	Sagittal standing	Sagittal standing extension	The level of significance of the analyzed variable between sagittal standing and sagittal standing extension
*kTH*				
0.002	57.9 (13.7)	43.3 (17.4)	35.5 (9.9)	0.001
*kLL*				
–	25.4 (11.4)	22.1 (12.2)	34.8 (12.3)	0.0002
*Pelvic inclination*				
0.0002	66.9 (20.9)	8.3 (3.8)	− 6.7 (17.4)	0.003
*Spine length*				
0.02	603.6 (122.7)	561.9 (85.5)	526.3 (98.1)	0.004

*kLL* lumbar lordosis angle, *kTH* thoracic kyphosis angle

The Spearman’s rank-order correlation between somatic parameters, markers, and qualities of the body posture is presented in Table 5. The results indicate moderate to large statistically significant relationships, especially regarding the spine length and somatic variables. The statistical analysis demonstrated a tendency to the interpenetration of relationships between variables that characterize (a) body structure and (b) function of the spine and musculoskeletal pain. Furthermore, the statistical analysis showed some moderate to large relationship between NMQ-7 (shoulders) and SL (R = 0.6; *p* < 0.05) and between SL and BH (R = 0.9; *p* < 0.01). Simultaneously, BH showed a statistically significant relationship with NMQ-7 (shoulders) (R = 0.5; *p* < 0.05). Similar statistically significant correlations were found between pelvic inclination and NMQ-7 (shoulders and lower back) (R = 0.5; *p* < 0.05/R = 06; *p* < 0.01). Moreover, pelvic inclination had a large negative correlation with trunk fat mass (R = − 0,6; *p* < 0.05) (Table 5).

**Table 5:** Qualities and indicators of the body build and posture and the prevalence of musculoskeletal pain (NMQ-7)—Spearman’s rank-order correlation

NMQ-7	R-value NMQ-7 versus SP (*p* < 0.01⁕⁕. *p* < 0.05⁕)	Somatic parameters (SP)	R-value SP versus ChS (*p* < 0.01⁕⁕. *p* < 0.05⁕)	Characteristics of the spine (ChS)	R-value ChS versus NMQ-7 (*p* < 0.01⁕⁕. *p* < 0.05⁕)	NMQ-7
×	×	BM (kg)	0.7**	SL	×	×
Shoulders	0.5*	BH (cm)	0.9**	SL	0.6*	Shoulders
			×	SLe	0.5*	
×	×	BAI (%)	− 0.6**	SL	×	×
×	×	WC (cm)	0.6**	SL	×	×
×	×	HC (cm)	0.5**	kTH in SSf	×	×
Elbows	0.6**	FT (%)	− 0.6*	PI	×	×
×	×	×	×	PIe	0.5*	Shoulders
					0.6**	LBP
×	×	×	×	Variability of kTH in SS versus SSe	− 0.5*	Wrists
					− 0.5*	Knees
×	×	×	×	kTH in SSe	0.5*	Neck
×	×	×	×	kLL in SSf	− 0.5*	Sum of the NMQ-7

*SP* somatic parameters, *FT* trunk fat mass, *ChS* characteristics of the spine, *SL* spine length, *SLe* spine length in sagittal standing extension, *kTh* the angle of thoracic kyphosis, *SSf* sagittal standing flexion, *SS* sagittal standing, *SSe* sagittal standing extension, *PI* pelvic inclination, *PIe* pelvic inclination extension, *kLL* the angle of lumbar lordosis, *LBP* low back pain

## Discussion

Among different types of motor impairments, lower limbs amputations seem to significantly impact body biomechanics [24, 25]. The aim of the study was to identify the impact of internal compensatory mechanisms on selected aspects of body structure and function in elite sitting volleyball players. The main finding of this study was that both trunk fat mass and BAI are the main determinants of musculoskeletal pain as it was found that they significantly affected the spine length and pelvic inclination. The results of the present study confirmed our initial hypothesis as they showed that the abovementioned components may induce internal compensations in the upper body segments in Para athletes, thus contributing to pain. Moreover, we found statistically significant relationships between the depth of thoracic kyphosis angle and (a) sagittal standing position and (b) sagittal standing flexion position, which may suggest that the thoracic segment of the spine is characterized by high mobility as a result of internal compensation for decreased mobility in the lumbar segment.

Several reasons might explain the variability of the angles of thoracic kyphosis, lumbar lordosis, and pelvic inclination that were observed in the study. Firstly, in a balanced spine, thoracic kyphosis and lumbar lordosis are intrinsically related. Consequently, one curvature responds to the disturbance in the other, while the pelvic position strongly interacts with the spinal shape by controlling the sagittal balance between the abovementioned curvatures [4]. As the study participants had lower limb impairment, the intrinsic spinal balance was disturbed. Hendershot et al. [10] indicated that changes in postural control and the area of spinal stability may be a result of the adaptations in functional tissues and/or neuromuscular response to repetitive exposure to abnormal gait and movement after lower limb/limbs amputations. Taking this into account, it can be postulated that in sitting volleyball players, the mechanism of internal compensation, activated by lower limb impairment, contributed to decreasing the angle of lumbar lordosis as the majority of the participants had lumbar hypolordosis. Moreover, the differences in thoracic kyphosis angle and mobility may also affect body biomechanics because of the lower limb impairment.

Sitting volleyball players were characterized by an excessive trunk fat mass and inappropriate BAI, which were found to impact on decreasing pelvic inclination and spine length that simultaneously contributed to shoulders pain. Those results are consistent with the studies by Brandt et al. [11], who indicated that lower limbs amputations may impact muscular changes in hips, and consequently affect pelvic inclination. In addition, the study conducted by Mirbagheri et al. [26] and Zwierzchowska et al. [15, 16] on the able-bodied population found that inappropriate BAI contributes to the deepening of the lumbar lordosis and LBP. Interestingly, our study revealed that the angle of lumbar lordosis significantly contributed to the prevalence of musculoskeletal pain. Given the above, there is a reason to believe that both individual characteristics of Para athletes and lumbar lordosis angle may be determining factors in inducing musculoskeletal pain due to the body’s intrinsic compensation.

To the best of the authors’ knowledge, the present study is the first to analyze the impact of spine length and pelvic inclination on musculoskeletal pain in Para athletes. Despite the large body of evidence on musculoskeletal complaints in able-bodied volleyball players, the data on sitting volleyball players are very limited. However, neck pain and LBP were indicated as the most common problems in elite able-bodied volleyball players by several authors [27–29], which is consistent with the findings of our study. Furthermore, the aforementioned painful areas simultaneously showed strong relationships with skeletal parameters (thoracic kyphosis angle, pelvic inclination). According to Gaweł et al. [4], the angles of thoracic kyphosis and lumbar lordosis are determinants of LBP in sitting volleyball players. Moreover, Mizoguchi et al. [30] found that decreased flexibility of the hip and shoulder flexors of the dominant limb may cause LBP. Taking this into account, decreased spine length and lumbar hypolordosis that were found in the Para athletes could cause LBP.

## Limitations

Our study has several limitations that need to be acknowledged. The study participants were elite Para athletes (the entire men’s and women’s Polish national team) and therefore the study groups were not large. However, it should be mentioned that there are few Para athletes at an elite level that could be included in the examinations. Furthermore, the studied group included Para athletes from one Paralympic sport. We examined more male sitting volleyball players, which makes any generalization impossible. Thus, future studies should be extended to include more Para athletes from various Paralympic sports, of both genders, and different types of impairment to enable generalization. Such studies could provide important data to help improve sports performance of Para athletes by minimizing the disadvantageous effects in body biomechanics [24, 25] because of intrinsic compensatory mechanisms and the proper prevention of musculoskeletal pain.

## Conclusions

1. The current study provides novel insights into the monitoring of the training process of Para athletes with lower limb impairment as it indicates the importance of internal compensatory mechanisms and provides important data on the prevalence and location of musculoskeletal pain.
2. Based on the main findings of this study, it can be concluded that trunk fat mass induces internal compensatory mechanisms to maintain optimal pelvic inclination and sagittal balance of the spine. In this light, athletes and coaches are advised to include strength exercises for core, pelvic floor muscles, and gluteus muscles to improve biomechanics and body movement abilities and to decrease internal compensatory mechanisms of the body.
3. Moreover, as the level of pelvic mobility was found to be the factor that may determine musculoskeletal pain in Para athletes with lower limb impairment, athletes and coaches should consider performing exercises to improve pelvic girdle mobility.

## Data Availability

The data collected and analyzed during the current study are available from the corresponding author on reasonable request.

## Abbreviations

BMI: Body mass index
BAI: Body adiposity index
LBP: Low back pain
MD: Minimal disability
SD: Standard deviation
BH: Body height
BM: Body mass
HC: Hip circumference
WC: Waist circumference
TK: Thoracic kyphosis
LL: Lumbar lordosis
PV: P-value
kTH: The angle of thoracic kyphosis
kLL: The angle of lumbar lordosis
NMQ = 7: Nordic Musculoskeletal Questionnaire from the last seven days
FT: Trunk fat mass
SP: Somatic parameters
ChS: Characteristics of the spine
SL: Spine length
SLe: Spine length in sagittal standing extension
SSf: Sagittal standing flexion
SS: Sagittal standing
SSe: Sagittal standing extension
PI: Pelvic inclination
Pie: Pelvic inclination extension
